# Editorial: Mycoviruses and Related Viruses Infecting Fungi, Lower Eukaryotes, Plants and Insects

**DOI:** 10.3389/fmicb.2021.798598

**Published:** 2021-11-25

**Authors:** Hiromitsu Moriyama, Ioly Kotta-Loizou, Kook-Hyung Kim, Jiatao Xie

**Affiliations:** ^1^Laboratory of Plant Pathology, Graduate School of Agriculture, Tokyo University of Agriculture and Technology, Fuchu, Japan; ^2^Department of Life Sciences, Faculty of Natural Sciences, Imperial College London, London, United Kingdom; ^3^Department of Agricultural Biotechnology, College of Agriculture and Life Sciences, Plant Genomics and Breeding Institute, Research Institute of Agriculture and Life Sciences, Seoul National University, Seoul, South Korea; ^4^State Key Laboratory of Agricultural Microbiology, Huazhong Agricultural University, Wuhan, China

**Keywords:** mycovirus, RNA virus, virus transmission, virus-host interactions, virus evolution

The Frontiers Research Topic “*Mycoviruses and Related Viruses Infecting Fungi, Lower Eukaryotes, Plants and Insects*” was initiated in the context of *Frontiers in Microbiology* and *Frontiers in Plant Pathology*, covering three sections: (1) Virology, (2) Microbe and Virus Interactions with Plants, and (3) Plant Pathogen Interactions. This reflects the importance of mycovirus-host interplay in phytopathogenic fungi; however, the final article collection is not limited to mycovirus infections in plant pathogens. Our Research Topic accommodates 15 high-quality Original Research manuscripts covering a range of topics, including the discovery and characterization of double-stranded (ds) RNA and single-stranded (ss) RNA viruses, the study of the distribution, transmission, and evolution of viruses in fungal populations, and the effects of viruses on their fungal hosts.

*Chrysoviridae, Hypoviridae, Partitiviridae*, and *Reoviridae* are well-studied families that accommodate mycoviruses. Telengech et al. reported the presence of 20 new members of the *Partitiviridae* family in the plant pathogen *Rosellinia necatrix*, causative agent of dematophora root rot. Subsequently, transfection experiments revealed differential RNA accumulation and symptomatology in the natural host, *R. necatrix*, as compared to a heterologous host, the chestnut blight fungus *Cryphonectria parasitica*. Similarly, Yang et al. investigated the effects of Cryphonectria parasitica hypovirus 1 and Mycoreovirus 1, both originally derived from *C. parasitica*, on the heterologous host *Valsa mali*, the causative agent of apple dieback, illustrating potential for biological control applications but also highlighting the complex interactions between the two viruses and the host antiviral RNA silencing machinery. Kashif et al. linked the partial phenotypic recovery of a hypovirulent isolate of the forest pathogen *Heterobasidion*, causative agent of Heterobasidion root rot, infected with a member of *Partitiviridae* with alterations in the virus titer and host gene expression. Owashi et al. investigated the distribution patterns of three different viruses, including members of the families *Partitiviridae* and *Chrysoviridae*, in the rice blast fungus *Magnaporthe oryzae* in Japan.

*Polymycoviridae* is a newly established family of non-conventionally encapsidated dsRNA viruses. Three novel members of the family were reported: Penicillium janthinellum polymycovirus 1 (PjPmV-1), Beauveria bassiana polymycovirus 3 (BbPmV-3), and Aspergillus fumigatus polymycovirus 1M (AfuPmV-1M). Sato et al. confirmed that the PjPmV-1 proline-alanine-serine rich protein (PASrp), a hallmark of polymycoviruses hypothesized to coat the dsRNA genome, has RNA binding properties and is capable of associating with nucleic acids in a non-sequence specific manner. Filippou et al. illustrated that BbPmV-1 and −3 mediate their effects on the insect pathogen and popular biocontrol agent *Beauveria bassiana* by interfering with host carbon and nitrogen metabolism. Takahashi-Nakaguchi et al. established that AfuPmV-1 causes hypovirulence to the human pathogen *Aspergillus fumigatus* in an immunosuppressed mouse infection model.

*Botourmiaviridae* is another recently established family, accommodating positive-sense ssRNA viruses. Zhao et al. discovered a new member of the family in the plant pathogen *Fusarium oxysporum*, causative agent of Fusarium wilt. Fusarium oxysporum ourmia-like virus 1 is transmitted horizontally and causes hypovirulence to its host, showing potential for biological control applications.

Fusarium graminearum virus 1 (FgV1) is a positive-sense ssRNA virus related to the proposed family Fusariviridae that results in increased pigmentation, and decreased growth and virulence in the plant pathogen *Fusarium graminearum*, causative agent of Fusarium head blight. Yu and Kim determined the differential effects of FgV1 on its host in the absence of specific fungal transcription factors, providing insights on the molecular mechanisms underpinning the observed phenotypes. In parallel, Heo et al. investigated the evolution of FgV1, taking into account the viral genome sequences, the natural selection pressures acting on the viral proteins and the effects of FgV1 on its host.

In addition to the aforementioned filamentous fungi, yeasts also harbor mycoviruses and the killer yeast system in *Saccharomyces cerevisiae* has been extensively studied. Ramírez et al. reported the presence of a helper virus in the wine yeast *Torulaspora delbrueckii*; the helper virus is a member of the family *Totiviridae*, closely related to those in *S. cerevisiae*, and supports the replication cycle of the dsRNA element responsible for the killer phenotype.

Lichens are stable symbiotic associations between fungi and algae and/or cyanobacteria. Urayama et al. reported the presence of members of the family *Partitiviridae* in lichens.

Oomycetes, despite what their name suggests, are not considered fungi but fungus-like lower eukaryotic organisms. Uchida et al. discovered two positive-sense ssRNA viruses belonging to the family *Endornaviridae* in the plant pathogen *Phytophthora*. Both endornaviruses were shown to affect host hyphal growth, zoosporangium formation and fungicide resistance. Botella et al. reported for the first time the presence of negative-sense ss RNA viruses of the order *Bunyavirales* in marine oomycetes of the genus *Halophytophthora*.

Finally, Fujita et al. discovered *Partitiviridae*-like viruses in the oriental tea trotix moth and pest of tea plants, *Homona magnanima*. These viruses, designated as Osugoroshi viruses, are responsible for the observed phenomenon of late male killing that leads to female-biased sex ratios in *H. magnanima*.

As Associate Editors, we would like to take this opportunity to acknowledge all the contributing authors who chose our Research Topic “*Mycoviruses and Related Viruses Infecting Fungi, Lower Eukaryotes, Plants and Insects*” as a vehicle for sharing their fascinating work on mycoviruses.

## Author Contributions

IK-L wrote the manuscript. All authors contributed to the article and approved the submitted version.

## Conflict of Interest

The authors declare that the research was conducted in the absence of any commercial or financial relationships that could be construed as a potential conflict of interest.

## Publisher's Note

All claims expressed in this article are solely those of the authors and do not necessarily represent those of their affiliated organizations, or those of the publisher, the editors and the reviewers. Any product that may be evaluated in this article, or claim that may be made by its manufacturer, is not guaranteed or endorsed by the publisher.

